# Retained Placenta in Miscarriage—How Shall We Treat Such Cases?*1. Read before the Tennessee State Medical Society April 15, 1891.

**Published:** 1891-08

**Authors:** A. J. Swaney

**Affiliations:** Gallatin, Tenn.


### RETAINED PLACENTA IN MISCARRIAGE — HOW SHALL WE TREAT SUCH CASES? *

BY A. J. SWANEY, M. D., GALLATIN, TENN.

The dangers from retained placenta in miscarriage are hemorrhage and septicemia. When the delivery of the placenta is prolonged, ought we still to abstain, ought we to wait or to interfere actively, in order to forestall these dangers which almost infallibly will result and further interfere at a time when it is far easier, than later when we may be forced to action? Such is the problem, the answer to which divides obstetricians into two opposing forces. One insists on active intervention to deliver the placenta as soon as possible and thus avert these dangers. The other having a deep faith in the powers of nature only allows interference when these complications become serious. Those who favor active interference are: Tyler, Smith, Murray, Simpson, Leishman, Munde, Grandin and many others. The reasons given for active interference are the frequency of these dangers in prolonged delivery of the placenta.

The almost constant possibility of manual extraction which at once assures the woman's safety from the dangers of hemorrhage and septic poisoning. Simpson, Viet and Munde are, perhaps, the most active partisans for interference. Simpson directs that if the cervix is dilated or patent, to act at

* 1. Read before the Tennessee State Medical Society April 15, 1891.

once; if it is not dilated he dilates it at once. The woman is then anaesthetized, the uterus depressed as much as possible by the external hand and with the index finger of the other hand he removes the placenta and membrane.

If he cannot sufficiently depress the uterus with the hand, he does not hesitate to forcibly drag it down by a double tenaculum fixed in the cervix. Munde and Dr. Grandin, of New York, go still further and curette the cavity of the uterus with special instruments made for the loosening of adherent placenta and its removal from the uterus. These curettes have no cutting edge and are applicable to cases where there is a large mass to remove and where, in consequence, nearly always the cervical canal is open and will admit them. When dealing with shreds, and the os is less patent, the dull curette of Thomas will answer every purpose. They place the woman in the left lateral position and the removal is through a Sims speculum. Dr. E. H. Grandin, of New York, then directs, after the removal of the placenta, that the cavity should be carefully dried by a cotton applicator and tamponed by means of a slide applicator, the cotton on which has been saturated with the compound tincture of iodine. The compound tincture of iodine is used as a gentle styptic and disinfectant.

The authorities who counsel waiting for serious complications before interfering, are just as many. We mention Ramsbotham, Davis, Burns, Fleetwood, Churchill, Grailey, Hewitt and many others. Charpentier, in his Cyclopœdia of Obstetrics and Gynecology, says: "If the woman miscarries in two stages, if the fœtus has been expelled and the placenta remains, what is to be done? Usually nothing, nature can do the work; the placenta may remain for days before being expelled, whilst there are no complications; wait, at least, until the placenta is engaged in the cervix and detached from the uterus, and then extract."

If the placenta is not engaged and the cervix is closed, wait, and in case of hemorrhage, tampon and give ergot, never the ergot alone. If the placenta, still adherent, and is in part engaged in the cervix, give ergot, for the cervix cannot retract, since its canal is filled by the placenta. If the placenta is at the fundus and adherent, wait still in case there is no complication, interfere in case of accident; if it be hemorrhage, the tampon and ergot. If it be putrefaction of the placenta, recognize this and extract at once; we must not hesitate, but we must immediately extract the placenta, or the secundines, and this it is understood is all the more difficult the more completely the cervix has closed. If the cervix is permeable to the finger or instruments, the operation is easy. If closed, then we must dilate at once with sponge, branched steel dilators or with Barnes' bags. Dilatation once accomplished we must proceed to extraction, and this must be done by the finger, or by instruments, according to the case. He directs after the cervix has been dilated, and the woman on her back, to depress the uterus with the left hand as much as possible, and with the index finger of the right hand introduced into the cavity of the uterus, or as deep as possible, the adherent remnants are detached and brought away. If this does not suffice he resorts to instruments.

Septicemia being one of the dangers from putrefaction of the retained placenta, how are we to recognize this? The first symptom is fetor of the lochial discharge. The discharge further loses its normal character and diminishes in quantity, becoming in color black or deep brown. It is no longer bloody or sero sanguinolent, but is composed of reddish black detritus, the debris of the retained mass, involution ceases and the uterus becomes sensitive to pressure. At times tympanites supervenes with or without diarrhoea. The woman has chills, sometimes the chills are violent and single, at other times many, separated by intervals of one or two days. There is fever, the temperature rising to 104-5 deg., the pulse ranges to 120 or more, the temperature shows marked remissions, but the pulse remains high, and thus it may be day after day until the woman dies, or the fever may be continuous.

The general condition alters for the worse, the eyes are sunken, anorexia, vomiting and diarrhoea exists, the woman grows weaker, and, if we cannot suppress these symptoms, dies of septic poisoning.

Has the physician any business to allow a woman, with retained placenta, to enter such a state as this? Is he doing his duty to sit calmly waiting for the onset of sepsis? He knows what he ought to do in case of sepsis, but action then, no matter how prompt, may fail and the woman die of septicemia. Then as we cannot tell in any given case of retained placenta or secundines, whether or not sepsis may develop, which is the wise course to pursue? To sit calmly waiting for the approach of these dangers as advised by Charpentier, Ramsbotham and others, or to act promptly, as advised by Munde, Grandin, Simpson and others?

I believe with Grandin that active interference in the removal of the retained placenta is safe, easy and forthwith guarantees the woman against sepsis.

Active intervention does not mean unnecessary interference; nature is ever to be given a chance, but when we see that her efforts are futile, certainly it is but rational to assist her, and this should be done as directed by Munde, by placing the woman in the left lateral position, and with a dull wire curette, remove the placenta, or any part of the secundines that may remain, through a Sims speculum. This is far better and easier than the method advised by Simpson, of dragging or pressing down the uterus and introducing the finger into the uterine cavity. The uterine cavity should then be washed out with hot water, slightly carbolized, through a Janeson's uterine douche, and this should be repeated every six or eight hours until all fetors disappears from the lochial discharge. I am painfully aware of the fact that a large majority of physicians in the country adopt and practice the expectant or do-nothing plan, many being satisfied simply with the vaginal injections. It would doubtless be a surprise to many if we could ascertain the number of valuable lives that have been lost, and the amount of suffering entailed upon women from the neglect in removing the retained placenta. Who of us with much experience has not seen such cases?

Shall we give ergot in retained placenta? This is another practice which should be relegated to the past. Engleman says never give ergot until the uterine cavity is cleared. The contractility evoked by ergot differs notably from that which is peculiar to the uterus, it is a species of tetanic retraction, which, when it affects the cervix, not only does not cause dilatation, but produces rigidity. Ergot may then act directly opposite to the desired end, and by interfering with the dilatation of the cervix, shut up the uterine cavity.

Hemorrhage after miscarriage, even when we believe the placenta and secundines have been removed, invariably means retention of a part of the placenta or secundines. Profuse hemorrhage may occur for weeks from this cause, and in such cases we should boldly explore the uterine cavity and remove any offending matter that may be present. In the first twelve weeks of pregnancy the dangers from hemorrhage and septicemia are not so great, and the expectant plan is more justifiable. After the third month it is criminal negligence to wait and subject a woman to the dangers arising from retained placenta when she can be relieved by an operation which, if properly done, can do no harm, and spare her the risk of hemorrhage and septic poisoning.

Again with Munde, Grandin, Simpson and others, I will say, the early removal of the secundines is easy and safe, and forthwith guarantees the woman against the dangers of hemorrhage and sepsis.

